# Association between atherogenic dyslipidemia and muscle quality defined by myosteatosis

**DOI:** 10.3389/fendo.2024.1327522

**Published:** 2024-08-07

**Authors:** Hwi Seung Kim, Yun Kyung Cho, Myung Jin Kim, Eun Hee Kim, Min Jung Lee, Woo Je Lee, Hong-Kyu Kim, Chang Hee Jung

**Affiliations:** ^1^ Department of Internal Medicine, Chung-Ang University Gwangmyeong Hospital, Chung-Ang University College of Medicine, Gwangmyeong, Republic of Korea; ^2^ Department of Internal Medicine, Asan Medical Center, University of Ulsan College of Medicine, Seoul, Republic of Korea; ^3^ Asan Diabetes Center, Asan Medical Center, Seoul, Republic of Korea; ^4^ Department of Health Screening and Promotion Center, Asan Medical Center, University of Ulsan College of Medicine, Seoul, Republic of Korea

**Keywords:** dyslipidemia, skeletal muscle, sarcopenia, lipid, myosteatosis

## Abstract

**Background:**

Myosteatosis, ectopic fat accumulation in skeletal muscle, is a crucial component of sarcopenia, linked to various cardiometabolic diseases. This study aimed to analyze the association between dyslipidemia and myosteatosis using abdominal computed tomography (CT) in a large population.

**Methods:**

This study included 11,823 patients not taking lipid-lowering medications with abdominal CT taken between 2012 and 2013. Total abdominal muscle area (TAMA), measured at the L3 level, was segmented into skeletal muscle area (SMA) and intramuscular adipose tissue. SMA was further classified into normal attenuation muscle area (NAMA: good quality muscle) and low attenuation muscle area (poor quality muscle). NAMA divided by TAMA (NAMA/TAMA) represents good quality muscle. Atherosclerotic dyslipidemia was defined as high-density lipoprotein cholesterol (HDL-C) less than 40 mg/dL in men and 50 mg/dL in women, low-density lipoprotein cholesterol (LDL-C) greater than 160 mg/dL, triglycerides (TG) greater than 150 mg/dL, small dense LDL-C (sdLDL-C) greater than 50.0 mg/dL, or apolipoprotein B/A1 (apoB/A1) greater than 0.08.

**Results:**

The adjusted odds ratios (ORs) of dyslipidemia according to the HDL-C and sdLDL definitions were greater in both sexes in the lower quartiles (Q1~3) of NAMA/TAMA compared with Q4. As per other definitions, the ORs were significantly increased in only women for LDL-C and only men for TG and ApoB/A1. In men, all lipid parameters were significantly associated with NAMA/TAMA, while TG and ApoB/A1 did not show significant association in women.

**Conclusion:**

Myosteatosis measured in abdominal CT was significantly associated with a higher risk of dyslipidemia. Myosteatosis may be an important risk factor for dyslipidemia and ensuing cardiometabolic diseases.

## 1 Introduction

Sarcopenia, traditionally defined as low muscle mass, is associated with numerous cardio-metabolic disorders, including atherogenic dyslipidemia (1–3). However, sarcopenia is a more complex condition that cannot be fully explained by the loss of muscle mass (4, 5). Recently, it has gained more attention for its association with muscle strength (4). A lack of muscle strength, a key characteristic of sarcopenia, can be indirectly assessed through muscle quality, encompassing changes in skeletal muscle architecture, composition, and function (5). Indeed, muscle quality evaluated by muscle fat infiltration (called as myosteatosis), is emerging as an important measure of sarcopenia (4, 6–8). Myosteatosis, characterized by ectopic fat deposition in skeletal muscle, disrupts muscle strength and metabolism (6–8).

Although muscle biopsy is the gold standard for detecting myosteatosis, computed tomography (CT) is commonly used to evaluate muscle quality by measuring fat infiltration in muscle through muscle attenuation (7). In CT scans, high attenuation indicates a lower fat content and thus reflects healthy, good-quality muscle, whereas low attenuation represents a higher fat content and indicates unhealthy, poor-quality muscle (7). So far, myosteatosis defined by CT scan has been linked to conditions such as immobilization, metabolic syndrome, type 2 diabetes, coronary artery calcification, and nonalcoholic fatty liver disease (NAFLD) (9–11).

Previous studies, predominantly relying on dual-energy X-ray absorptiometry (DXA) to measure muscle mass for sarcopenia assessment, have explored the relationship between sarcopenia and atherogenic dyslipidemia (12, 13). Individuals with a low muscle mass index had a higher incidence and risk of atherogenic dyslipidemia (with low-density lipoprotein cholesterol, LDL-C, levels higher than the individual risk factor-based target) than those with a normal muscle mass index (12). Similarly, elderly Asian men with low skeletal muscle mass measured by DXA exhibited a higher prevalence and risk of atherogenic dyslipidemia and an overall worse lipid profile than those with normal muscle mass (13).

However, research on the association between dyslipidemia and muscle quality beyond muscle mass is lacking and inconsistent. In a study of Afro-Caribbean men, calf muscle adiposity was positively associated with low-density lipoprotein cholesterol (LDL-C) and inversely associated with high-density lipoprotein cholesterol (HDL-C) (14). The Multi-Ethnic Study of Atherosclerosis demonstrated a positive correlation between total abdominal muscle density and total cholesterol; however, no significant associations were found with LDL-C, HDL-C, or triglycerides (TG) (15). Given the heterogeneity in myosteatosis markers and study populations, findings on the association between myosteatosis and lipid levels are inconsistent. Therefore, we aimed to reevaluate the relationship between myosteatosis and atherogenic dyslipidemia in a large population using CT-measured muscle attenuation.

## 2 Materials and methods

### 2.1 Study population

**Figure 1:**
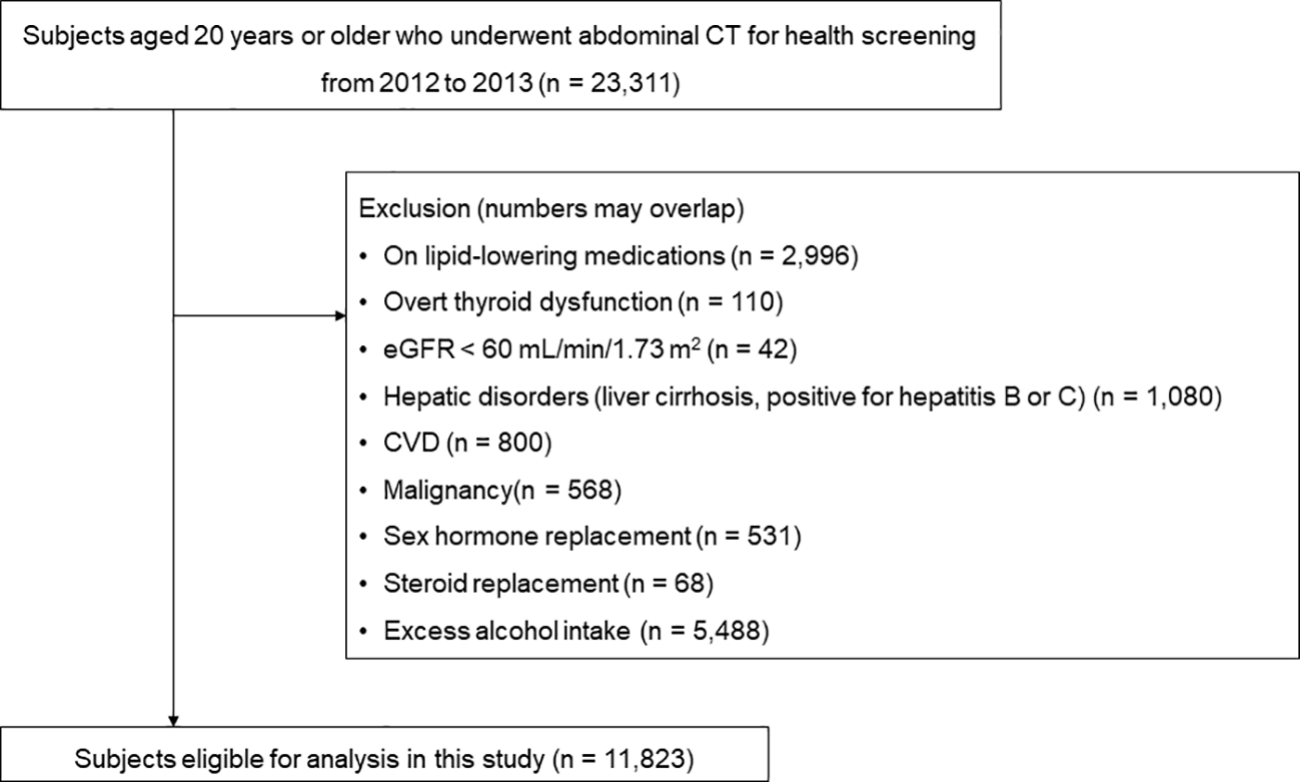
Selection process of the study population.

A total of 23,311 subjects who had undergone abdominal CT scans as part of routine health check-ups at the Health Screening and Promotion Center of Asan Medical Center (Seoul, Republic of Korea) between January 2012 and December 2013 were identified. Patients meeting one or more of the following criteria were excluded: those on lipid-lowering medications (n = 2,996), those with overt thyroid dysfunction (n = 110), and those with chronic renal insufficiency (estimated glomerular filtration rate < 60 mL/min/1.73 m^2^) (n = 42). Furthermore, subjects with hepatic disorders, cardiovascular disease, malignancy, or those currently taking glucocorticoids or hormone replacement therapy, as well as those with excessive alcohol intake (> 30 grams/day in men; > 20 grams/day in women), were excluded. Finally, 11,823 subjects were included in the analysis (**Figure 1**).

All participants completed a questionnaire on their medical and surgical history, medications, and health behaviors such as smoking, drinking, and exercise. Smoking behavior was categorized into three groups: current, past, and never smokers. Drinking habits were quantified in grams/day based on the alcohol content of the beverage, frequency of drinking, and amount consumed. Regular exercise was defined as 30 minutes of moderate-intensity aerobic exercise five days a week, 20 minutes of vigorous-intensity aerobic exercise three days a week, or resistance exercises 3 days a week. Hypertension was defined as having a systolic and/or diastolic blood pressure (BP) of 140/90 mmHg or higher or taking antihypertensive medication. Diabetes mellitus was diagnosed if any of the following criteria were met; fasting plasma glucose (FPG) level ≥ 126 mg/dL (7.0 mmol/L), glycated hemoglobin level (HbA1c) ≥ 6.5%, or current use of anti-diabetic medication.

This study adhered to the ethical guidelines outlined in the Declaration of Helsinki and Korea Good Clinical Practice. Written informed consent was obtained from all subjects. The institutional review board of Asan Medical Center approved this study (No. 2020-0343).

### 2.2 Measurements

Height and weight were measured while subjects were wearing light clothing without shoes. Body mass index (BMI) was calculated as weight in kilograms divided by the square of height in meters. Waist circumference (WC, cm) was measured midway between the costal margin and the iliac crest at the end of a normal expiration. BP was measured on the right arm after a rest ≥5 min, using an automatic manometer with an appropriate cuff size.

After overnight fasting, early morning blood samples were drawn from the antecubital vein into vacuum tubes and subsequently analyzed at a central, certified laboratory in Asan Medical Center. Measurements included the concentrations of uric acid, fasting glucose, high-sensitive C-reactive protein (hsCRP) and lipid parameters including, apolipoprotein B (apoB) and apolipoprotein A1 (apoA1).

Fasting total cholesterol (TC), HDL-C, LDL-C, TG and uric acid were measured by an enzymatic colorimetric method using a Toshiba 200FR Neo (Toshiba Medical System Co., Ltd., Tokyo, Japan). Serum apoB and apoA1 levels were measured by a turbidometric method using Cobas Integra C-6000 analyzer (Roche Diagnostics, Basel, Switzerland). HsCRP was measured by an immunoturbidimetric method (Toshiba). FPG levels were measured via an enzymatic colorimetric method using a Toshiba 200 FR autoanalyzer (Toshiba). Ion-exchange high-performance liquid chromatography (Bio-Rad Laboratories, Inc., Hercules, CA, USA) was used to measure HbA1c levels. All enzyme activities were measured at 37°C.

### 2.3 Definition of atherogenic dyslipidemia

Atherogenic dyslipidemia was defined as meeting at least one of the following three criteria, as per the guidelines of the National Cholesterol Education Program Expert Panel on Detection, Evaluation, and Treatment of High Blood Cholesterol in Adults (Adult Treatment Panel III) (16): low HDL-C < 40 mg/dL in men and < 50 mg/dL in women, high LDL-C ≥ 160 mg/dL, or high TG ≥ 150 mg/dL.

In addition to the classic dyslipidemia criteria, calculated small dense LDL-C (sdLDL-C) was determined using the following equation: 0.580 (non–HDL-C) + 0.407 (dLDL-C) – 0.719 (cLDL-C) – 12.05, where dLDL-C and cLDL-C represent the directly measured and calculated LDL-C, respectively, based on the equation TC – HDL-C – TG/5 (17). Furthermore, apolipoprotein B (ApoB)/apolipoprotein A1 (ApoA1) was measured in 1585 subjects. ApoA1 constitutes HDL-C, while ApoB is in very-low-density lipoprotein, intermediate-density lipoprotein, LDL-C, and lipoprotein(a) (18). Thus, ApoB/ApoA1 represents the balance between proatherogenic and antiatherogenic lipoproteins (18). The sdLDL-C cutoff value of 50.0 mg/dL was used, as established using samples from the MultiEthnic Study of Atherosclerosis (MESA) and was based on the 75th percentile value of sdLDL-C for normolipidemic and dyslipidemic subjects who showed no sign of coronary heart disease or diabetes mellitus at baseline (19). For ApoB/A1, 0.80 was used as the cutoff, as suggested in previous studies (20, 21).

### 2.4 CT image collection

Abdomen and pelvis CT scans were conducted using the Somatom Definition scanner (Siemens Healthineers, Erlangen, Germany), Discovery CT750 HD scanner (GE Healthcare, Milwaukee, WI, USA), or LightSpeed VCT scanner (GE Healthcare). All CT examinations were performed with the following parameters: 120 kVp; automated dose modulation (CareDose 4D, Siemens Healthineers; automA and smartmA, GE Healthcare); matrix 512 × 512; and collimation of 0.625 mm. Image data were reconstructed with a slice thickness of 5 mm using the filtered back-projection technique and a soft tissue reconstruction algorithm (B30f kernel; Siemens Healthineers; Standard kernel, GE Healthcare). For contrast enhancement, 100–150 mL of iopromide (Ultravist 370 or Ultravist 300; Bayer Schering Pharma, Berlin, Germany) was intravenously administered using an automatic power injector.

### 2.5 Assessment of body composition and myosteatosis

Body composition was evaluated using automated artificial intelligence software developed with a fully convolutional network segmentation technique. Since muscle mass, as measured by the lumbar 3rd vertebra (L3) CT, is strongly correlated with whole-body muscle mass (22), the software was programmed to automatically select the L3 vertebrae inferior endplate level. The selected CT images were then segmented automatically to generate the boundaries of the total abdominal muscle area (TAMA), visceral fat area (VFA), and subcutaneous fat area (SFA). All muscles within the selected axial images (including the psoas, para-spinal, transversus abdominis, rectus abdominis, quadratus lumborum, and internal and external obliques) were encompassed by TAMA. An image analyst and a radiologist, blinded to the clinical information, reviewed all selected CT images and verified the segmented areas.

TAMA was categorized based on CT density for myosteatosis measurement: (1) normal attenuation muscle area (NAMA, +30 to +150 Hounsfield Unit [HU]), representing nonfatty muscle with minimal intramuscular fat; (2) low attenuation muscle area (LAMA, −29 to +29 HU), representing fatty muscles with intramuscular lipid deposition; and (3) intermuscular adipose tissue (IMAT, −190 to −30 HU), representing visible fat tissue located between muscle groups and muscle fibers (**Supplementary Figure S1**) (23). The skeletal muscle area (SMA, −29 to +150 HU) included NAMA and LAMA. The NAMA/TAMA ratio was calculated by dividing NAMA by TAMA and multiplying by 100. VFA and SFA were also evaluated based on specific fat tissue thresholds (−190 to −30 HU).

### 2.6 Statistical analysis

Statistical analyses were performed for each sex due to the differences in muscle mass and attenuation value (6, 24). Continuous variables with normal distributions are shown as mean ± standard deviation, while those with skewed distributions are shown as median and interquartile range. Categorical variables are expressed as numbers (%). One way analysis of variance was applied to calculate the statistical significance among the quartile groups (**Tables 1**, **2**), while student’s t-test and chi-square test were performed to compare two groups (**Supplementary Table S2**). The sex-specific quartiles (Q1−Q4) of the NAMA/TAMA index were applied as the marker for myosteatosis throughout our analyses. The quartile ranges of the NAMA/TAMA index are presented in **Supplementary Table S1**. Multiple logistic regression analyses were performed to analyze the odds ratios (ORs) and 95% confidence intervals for dyslipidemia according to HDL-C, LDL-C, TG, sdLDL-C, and ApoB/A1 definitions. The ORs were adjusted for age, sex, smoking status, alcohol consumption, regular exercise, hypertension, diabetes mellitus, and VFA/SFA ratio. Menopausal status was also adjusted in females. Multiple linear regression analysis with the NAMA/TAMA index as a continuous variable was also performed. All statistical analyses were performed using SPSS software version 21.0 for Windows (IBM, Inc., Armonk, NY, USA). P-values of < 0.05 were considered statistically significant.

**Table 1:** Comparison of baseline characteristics and CT measurements between the quartiles of the NAMA/TAMA index in males.

	Q1 (n = 1,606)	Q2 (n = 1,613)	Q3 (n = 1,606)	Q4 (n = 1,609)	P-value
**Dyslipidemia, n (%)**	780 (48.6)^a^	746 (46.2)^a,b^	684 (42.6)^b,c^	618 (38.4)^c^	<0.001
Anthropometric data					
Age, years	57.2 ± 9.5	53.9 ± 8.4	52.3 ± 8.6	49.3 ± 8.6	<0.001
Height, cm	170.8 ± 6.0	170.8 ± 5.8	170.5 ± 5.6	170.3 ± 5.7	0.073
Weight, kg	75.0 ± 11.3	71.7 ± 9.0	70.1 ± 8.4	67.1 ± 8.5	<0.001
BMI, kg/m^2^	25.7 ± 3.1	24.5 ± 2.6	24.1 ± 2.4	23.1 ± 2.5	<0.001
WC, cm	91.7 ± 8.1	87.7 ± 6.5	85.7 ± 6.3	81.9 ± 7.0	<0.001
SBP, mmHg	126.2 ± 13.7	124.4 ± 12.7	123.1 ± 12.9^a^	121.9 ± 12.5^a^	<0.001
DBP, mmHg	80.4 ± 10.5^a^	79.8 ± 9.9^a^	78.8 ± 10.1^b^	78.0 ± 9.8^b^	<0.001
Skeletal muscle mass, kg	31.3 ± 4.2^a^	31.2 ± 3.7^a,b^	31.2 ± 3.6^a,b^	30.8 ± 3.6	0.003
Body fat mass, kg	19.0 ± 6.3	16.1 ± 4.5	14.7 ± 4.0	12.4 ± 4.1	<0.001
Biochemical data					
Fasting glucose, mg/dL	103.3 ± 19.6	101.5 ± 18.6	99.7 ± 16.9^a^	98.4 ± 17.2^a^	<0.001
HbA1c, %	5.8 ± 0.8	5.7 ± 0.7	5.6 ± 0.6	5.5 ± 0.6	<0.001
Total cholesterol, mg/dL	194.4 ± 33.9	196.2 ± 32.4	196.3 ± 33.1	195.2 ± 33.5	0.308
Triglycerides, mg/dL	117 (84–161)^a^	115 (83–159)^a,b^	109 (80–151)^a,b,c^	102 (74–145)^c^	<0.001
HDL-C, mg/dL	48.4 ± 11.6^a^	49.4 ± 12.3^a,b^	50.0 ± 11.9^b^	52.6 ± 13.3	<0.001
LDL-C, mg/dL	126.6 ± 30.0^a^	127.7 ± 28.6^a^	128.1 ± 29.1^a^	125.4 ± 29.5^a^	0.049
AST, IU/L	26 (21–32)^a^	26 (21–31)^a,b,c^	25 (21–31)^a,b,c^	25 (21–30)^c^	0.031
ALT, IU/L	24 (18–33)^a^	24 (18–33)^a,b^	23 (18–33)^a,b^	22 (17–30)	<0.001
hsCRP, mg/dL	0.07 (0.04–0.15)	0.06 (0.03–0.12)^a^	0.05 (0.03–0.11)^a^	0.04 (0.02–0.08)	<0.001
eGFR, mL/min/1.73 cm^2^	94.4 ± 16.2^a^	93.4 ± 15.1^a,b^	92.2 ± 14.3^b,c^	92.2 ± 14.4^b,c^	<0.001
Clinical data					
Current smoker, n (%)	485 (30.2)	495 (30.7)	505 (31.5)	535 (33.3)	0.099
Alcohol consumption, g/d	5.3 (0.9–12.4)	6.4 (1.1–13.8)	6.3 (1.2–11.7)	6.8 (1.4–12.1)	0.294
Regular exercise, n (%)	890 (55.5)^a^	936 (58.1)^a,b^	979 (61.1)^b,c^	991 (61.8)^b,c^	<0.001
Diabetes, n (%)	182 (11.3)	142 (8.8)^a^	116 (7.2)^a,b^	99 (6.2)^b^	<0.001
Hypertension, n (%)	492 (30.6)	348 (21.6)^a^	303 (18.9)^a^	218 (13.5)	<0.001
CT measurement data					
SMA, cm^2^	158.8 ± 23.0	161.7 ± 20.8^a^	163.5 ± 20.7^a,b^	164.2 ± 21.6^b^	<0.001
SMA/BMI	6.2 ± 0.7	6.6 ± 0.6	6.8 ± 0.6	7.1 ± 0.6	<0.001
NAMA, cm^2^	113.4 ± 19.0	128.6 ± 16.7	136.3 ± 17.4	143.9 ± 19.0	<0.001
NAMA/BMI	4.4 ± 0.7	5.2 ± 0.5	5.7 ± 0.5	6.2 ± 0.6	<0.001
LAMA, cm^2^	45.4 ± 11.1	33.1 ± 5.3	27.2 ± 4.1	20.3 ± 4.2	<0.001
LAMA/BMI	1.8 ± 0.3	1.3 ± 0.2	1.1 ± 0.1	0.9 ± 0.1	<0.001
NAMA/TAMA index	67.3 ± 6.3	76.7 ± 1.5	81.4 ± 1.2	86.5 ± 2.1	<0.001
VFA/SFA	1.2 ± 0.5	1.1 ± 0.4	1.1 ± 0.4	0.9 ± 0.4	<0.001

Data are presented as mean ± standard deviation or median (interquartile range, 1st–4th) unless otherwise indicated.

CT, computed tomography; NAMA, normal attenuation muscle area; TAMA, total abdominal muscle area; Q, quartile; BMI, body mass index; WC, waist circumference; SBP, systolic blood pressure; DBP, diastolic blood pressure; HbA1c, glycated hemoglobin; HDL-C, high-density lipoprotein cholesterol; LDL-C, low-density lipoprotein cholesterol; AST, aspartate aminotransferase; ALT, alanine aminotransferase; hsCRP, high-sensitivity C-reactive protein; eGFR, estimated glomerular filtration rate; SMA, skeletal muscle area; LAMA, low attenuation muscle area; VFA, visceral fat area; SFA, subcutaneous fat area.

^a,b,c^ Superscript letters imply a statistically insignificant difference in the post-hoc analysis between values indicated by the same superscript letter. Otherwise, the post-hoc analysis revealed significant differences between each group.

**Table 2:** Comparison of baseline characteristics and CT measurements between the quartiles of the NAMA/TAMA index in females.

	Q1 (n = 1345)	Q2 (n = 1351)	Q3 (n = 1348)	Q4 (n = 1345)	P-value
**Dyslipidemia, n (%)**	646 (48.0)	517 (38.3)^a^	482 (35.8)^a^	300 (22.3)	<0.001
Anthropometric data					
Age, years	57.9 ± 8.6	53.3 ± 7.5	51.5 ± 7.6	47.1 ± 7.2	<0.001
Height, cm	157.6 ± 5.4	158.5 ± 5.4^a^	158.4 ± 5.1^a^	159.4 ± 5.2	0.075
Weight, kg	61.1 ± 8.3	57.8 ± 7.2	55.3 ± 6.5	52.7 ± 6.1	<0.001
BMI, kg/m^2^	24.6 ± 3.1	23.0 ± 2.7	22.1 ± 2.4	20.7 ± 2.3	<0.001
WC, cm	84.9 ± 7.8	79.7 ± 6.9	76.7 ± 6.5	72.3 ± 6.3	<0.001
SBP, mmHg	122.9 ± 15.2	117.6 ± 14.5	115.5 ± 13.9	112.2 ± 13.2	<0.001
DBP, mmHg	75.9 ± 10.4	73.3 ± 10.6	72.1 ± 10.6	70.3 ± 10.3	0.727
Skeletal muscle mass, kg	21.6 ± 2.6	21.7 ± 2.4	21.5 ± 2.4	21.5 ± 2.3	<0.001
Body fat mass, kg	20.9 ± 5.7	17.6 ± 4.7	15.7 ± 4.2	13.0 ± 3.9	<0.001
Biochemical data					
Fasting glucose, mg/dL	100.1 ± 19.0	97.1 ± 15.9	95.1 ± 12.9	92.6 ± 11.3	<0.001
HbA1c, %	5.7 ± 0.7	5.6 ± 0.6	5.5 ± 0.5	5.4 ± 0.4	<0.001
Total cholesterol, mg/dL	206.3 ± 34.3^a^	203.3 ± 34.0^a,b^	201.7 ± 33.4^b^	192.4 ± 31.4	0.021
Triglycerides, mg/dL	95 (69–130)	85 (65–120)	82 (62–113)	71 (53–98)	<0.001
HDL-C, mg/dL	57.9 ± 14.0	60.6 ± 14.2	62.2 ± 14.7	66.0 ± 14.8	0.171
LDL-C, mg/dL	132.5 ± 30.8	128.2 ± 30.6^a^	126.1 ± 30.1^a^	115.5 ± 28.2	0.006
AST, IU/L	24 (20–29)^a^	23 (20–29)^a,b^	23 (19–28)^a,b^	22 (19–26)	0.001
ALT, IU/L	19 (15–25)	18 (14–24)^a^	16 (13–22)^a^	15 (12–20)	<0.001
hsCRP, mg/dL	0.06 (0.03–0.13)	0.04 (0.02–0.09)^a^	0.03 (0.02–0.07)^a,b^	0.02 (0.02–0.05)^b^	<0.001
eGFR, mL/min/1.73 cm^2^	101.8 ± 19.1^a^	101.8 ± 17.8^a^	100.4 ± 16.7^a^	100.9 ± 16.4^a^	<0.001
Clinical data					
Current smoker, n (%)	34 (2.5)^a^	37 (2.7)^a^	32 (2.4)^a^	52 (3.9)	0.004
Alcohol consumption, g/d	0.0 (0.0–1.2)^a^	0.4 (0.0–1.9)^a,b^	0.4 (0.0–1.9)^b,c^	0.8 (0.0–2.7)^c^	<0.001
Regular exercise, n (%)	691 (51.5)	745 (55.2)^a^	804 (59.8)^b^	763 (56.8)^a,b^	<0.001
Menopause, n (%)	1,095 (81.4)	888 (65.7)	749 (55.6)	459 (34.1)	<0.001
Diabetes, n (%)	106 (7.9)	62 (4.6)	34 (2.5)^a^	22 (1.6)^a^	<0.001
Hypertension, n (%)	319 (23.7)	189 (14.0)	130 (9.6)	78 (5.8)	<0.001
CT measurement data					
SMA, cm^2^	105.9 ± 13.3	107.5 ± 12.9^a^	107.3 ± 13.0^a^	108.1 ± 12.8^a^	0.592
SMA/BMI	4.3 ± 0.5	4.7 ± 0.5	4.9 ± 0.5	5.2 ± 0.6	<0.001
NAMA, cm^2^	69.0 ± 11.0	80.5 ± 9.8	85.6 ± 10.5	91.8 ± 11.1	0.001
NAMA/BMI	2.8 ± 0.5	3.5 ± 0.4	3.9 ± 0.4	4.4 ± 0.5	<0.001
LAMA, cm^2^	37.0 ± 8.2	26.9 ± 4.2	21.7 ± 3.2	16.3 ± 3.2	<0.001
LAMA/BMI	1.5 ± 0.3	1.2 ± 0.2	1.0 ± 0.1	0.8 ± 0.1	<0.001
NAMA/TAMA index	59.7 ± 6.9	71.2 ± 2.0	77.1 ± 1.5	83.3 ± 2.5	<0.001
VFA/SFA	0.6 ± 0.3	0.5 ± 0.2	0.5 ± 0.2	0.3 ± 0.2	<0.001

Data are presented as mean ± standard deviation or median (interquartile range, 1st–4th) unless otherwise indicated.

CT, computed tomography; NAMA, normal attenuation muscle area; TAMA, total abdominal muscle area; Q, quartile; BMI, body mass index; WC, waist circumference; SBP, systolic blood pressure; DBP, diastolic blood pressure; HbA1c, glycated hemoglobin; HDL-C, high-density lipoprotein cholesterol; LDL-C, low-density lipoprotein cholesterol; AST, aspartate aminotransferase; ALT, alanine aminotransferase; hsCRP, high-sensitivity C-reactive protein; eGFR, estimated glomerular filtration rate; SMA, skeletal muscle area; LAMA, low attenuation muscle area; VFA, visceral fat area; SFA, subcutaneous fat area.

^a,b,c^The superscript letters imply a statistically insignificant difference in the post-hoc analysis between values indicated by the same superscript letter. Otherwise, the post-hoc analysis revealed significant differences between each group.

## 3 Results

### 3.1 Baseline characteristics

Of the 11,823 subjects, 6,434 were men and 5,389 were women, among which dyslipidemia was present in 4,773 (40.4%). The baseline characteristics and CT measurements between the NAMA/TAMA index quartiles are presented in **Tables 1**, **2** for each sex. As the NAMA/TAMA quartile increased, the prevalence of dyslipidemia decreased in both male and female populations. The higher the NAMA/TAMA quartile, the younger the patients, with overall favorable metabolic profiles such as lower BMI, WC, BP, and FPG levels across both sexes. Myosteatosis indices were also the most favorable in the highest quartile group for both men and women. Male and female patients with dyslipidemia weighed more and had a larger BMI, WC, and body fat mass than those without dyslipidemia (**Supplementary Table S2**). Those without dyslipidemia showed greater value of NAMA/TAMA than those with dyslipidemia regardless of sex (**Supplementary Table S2**).

### 3.2 Risk of atherogenic dyslipidemia

**Figure 2:**
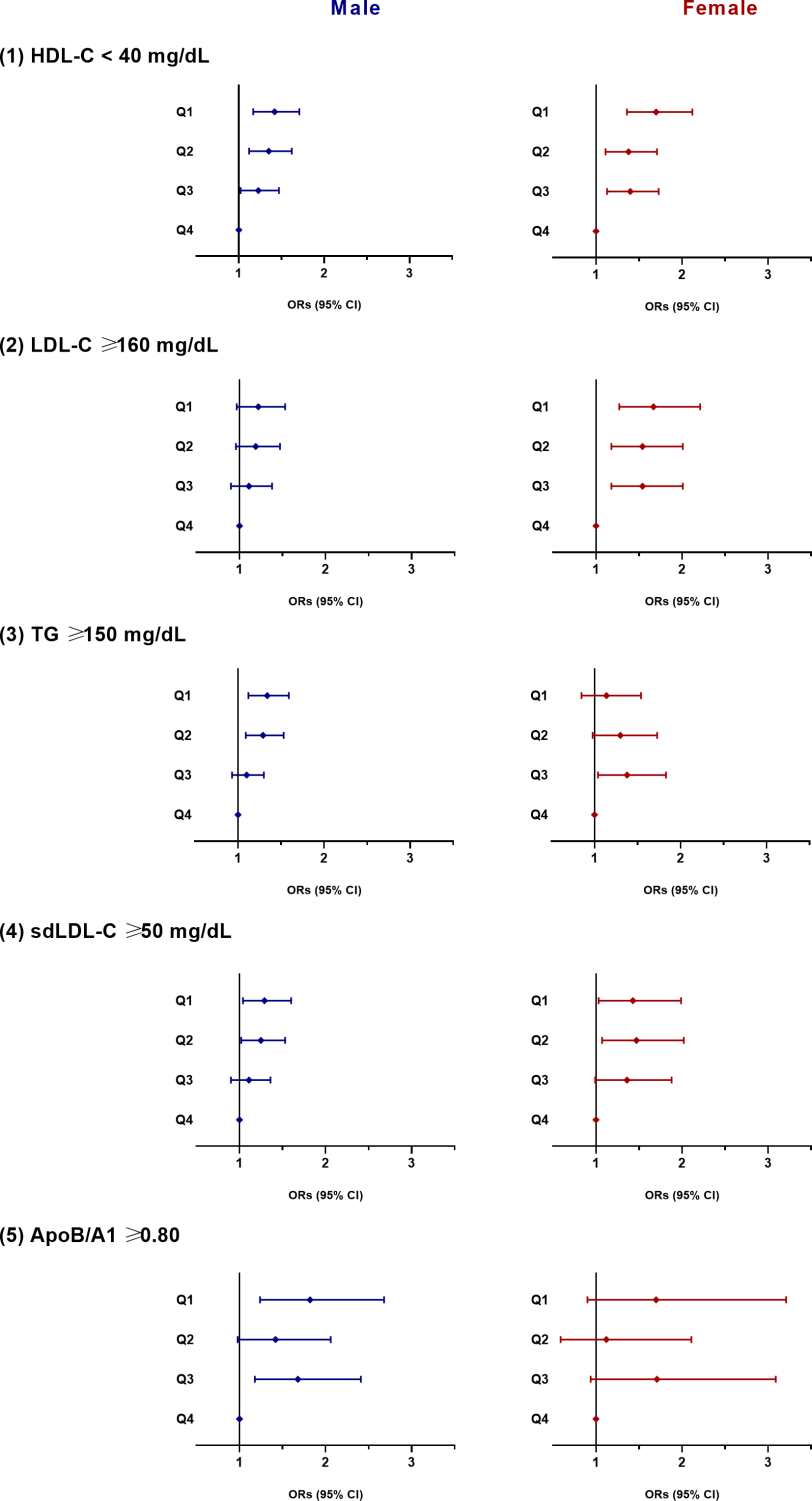
ORs for dyslipidemia according to the quartiles of the NAMA/TAMA index. ORs were adjusted for age, smoking, alcohol consumption, exercise, hypertension, diabetes, menopausal status (in female), and VFA to SFA ratio. OR, odds ratio; NAMA, normal attenuation muscle area; TAMA, total abdominal muscle area; Q, quartile; HDL-C, high-density lipoprotein cholesterol; LDL-C, low-density lipoprotein cholesterol; VFA, visceral fat area; SFA, subcutaneous fat area.

The risk of atherogenic dyslipidemia according to the quartiles of the NAMA/TAMA index for each sex are shown in **Figure 2**. In men, as the NAMA/TAMA quartile decreased, the ORs of dyslipidemia, according to the HDL-C, TG, sdLDL, and ApoB/A1 definitions, significantly increased (**Figure 2**). According to the LDL-C definition, the NAMA/TAMA quartiles in males were not significantly associated with a higher risk of dyslipidemia (**Figure 2**). The NAMA/TAMA index was positively associated with HDL-C and negatively associated with LDL-C, TG, sdLDL, and ApoB/A1, and all of the standardized beta values were statistically significant in the male population (**Table 3**).

**Table 3:** Multiple linear regression analysis of the lipid parameters.

	Male		Female	
	Standardized β	P value	Standardized β	P value
1) HDL-C				
NAMA/TAMA	0.069	< 0.001	0.080	< 0.001
Age	0.094	< 0.001	0.042	0.013
Smoking	-0.066	< 0.001	0.015	0.215
Alcohol	0.119	< 0.001	0.082	< 0.001
Exercise	0.249	< 0.001	-0.055	< 0.001
Hypertension	0.257	0.077	-0.008	0.532
Diabetes	0.384	< 0.001	-0.063	< 0.001
Menopausal status			0.031	0.031
VFA/SFA	0.267	< 0.001	0.267	< 0.001
2) LDL-C				
NAMA/TAMA	-0.070	< 0.001	-0.065	< 0.001
Age	0.149	< 0.001	0.075	< 0.001
Smoking	-0.030	0.012	-0.030	0.014
Alcohol	-0.006	0.613	-0.007	0.556
Exercise	0.008	0.513	0.010	0.426
Hypertension	-0.045	< 0.001	-0.043	0.001
Diabetes	-0.079	< 0.001	-0.079	< 0.001
Menopausal status			0.127	< 0.001
VFA/SFA	0.180	< 0.001	0.182	< 0.001
3) TG				
NAMA/TAMA	-0.035	< 0.001	-0.017	0.208
Age	-0.178	< 0.001	0.005	0.744
Smoking	0.120	< 0.001	0.042	< 0.001
Alcohol	0.017	< 0.001	-0.021	0.073
Exercise	0.077	0.059	0.044	< 0.001
Hypertension	0.086	< 0.001	0.041	0.001
Diabetes	0.046	< 0.001	0.067	< 0.001
Menopausal status			0.047	0.001
VFA/SFA	0.241	< 0.001	0.326	< 0.001
4) sdLDL-C				
NAMA/TAMA	-0.038	0.004	-0.056	<0.001
Age	-0.186	<0.001	0.042	0.019
Smoking	0.101	<0.001	0.012	0.344
Alcohol	-0.021	0.084	-0.037	0.004
Exercise	0.070	<0.001	0.021	0.099
Hypertension	0.019	0.113	-0.005	0.735
Diabetes	-0.052	<0.001	-0.033	0.012
Menopausal status			0.118	<0.001
VFA/SFA	0.230	<0.001	0.315	<0.001
5) ApoB/A1				
NAMA/TAMA	-0.098	0.002	-0.089	0.065
Age	-0.142	<0.001	-0.0789	0.158
Smoking	0.137	<0.001	-0.038	0.368
Alcohol	-0.094	0.002	-0.095	0.030
Exercise	0.081	0.008	0.051	0.220
Hypertension	0.020	0.518	-0.013	0.766
Diabetes	-0.112	<0.001	-0.086	0.044
Menopausal status			0.128	0.008
VFA/SFA	0.164	<0.001	0.300	<0.001

In women, a decrease in the NAMA/TAMA quartile resulted in higher ORs for dyslipidemia according to HDL-C, LDL-C, and sdLDL-C definitions (**Figure 2**). According to the TG and ApoB/A1 definitions, the ORs for dyslipidemia was not statistically significant in the female population (**Figure 2**). The NAMA/TAMA index was positively associated with HDL-C and negatively associated with LDL-C, TG, sdLDL-C, and ApoB/A1 in women (**Table 3**). However, the standardized beta values of TG and ApoB/A1 were not statistically significant (**Table 3**).

## 4 Discussion

This cross-sectional analysis of 11,823 patients who participated in routine health examinations showed that muscle quality assessed by the degree of myosteatosis was associated with the risk of atherogenic dyslipidemia. A greater NAMA/TAMA index, which indicates healthy low-fat muscle, was significantly associated with a lower risk of atherogenic dyslipidemia even after adjusting for age, health behaviors, and other ectopic fat distribution.

The value of myosteatosis is on the rise in the research field of sarcopenia. Myosteatosis allows the measurement of muscle microstructure, which determines muscle strength and metabolism (25, 26). Type 2 diabetes, NAFLD, and cardiovascular disease are some of the unfavorable clinical outcomes associated with myosteatosis (9–11). Patients with type 2 diabetes exhibited increased LAMA and decreased NAMA and NAMA/TAMA index (27). Additionally, those with higher LAMA and lower NAMA/TAMA were at a higher risk of NAFLD and liver fibrosis (11).

Myosteatosis is also associated with mortality. Increased myosteatosis determined by calf muscle density using CT was correlated with increased all-cause and cardiovascular mortality in older men, according to a 7.2-year longitudinal study (28). According to an 8.8-year follow-up study, myosteatosis measured in the thigh muscle using CT was associated with a higher mortality risk (29). Mortality risk decreased in patients with higher abdominal muscle density, highlighting muscle quality as a predictor of mortality (30).

Only a few studies have examined the associations between muscle density measured by CT and lipid levels. Miljkovic et al. demonstrated that calf muscle density was positively associated with LDL-C and negatively associated with HDL-C (14). These findings were additionally partly supported by a longitudinal study that revealed exercise-induced reductions in thigh IMAT were related to changes in HDL-C and LDL-C to larger, less atherogenic lipoprotein particles (31). However, Vella et al. later reported that increases in total cholesterol levels (not LDL-C, HDL-C, or TG) were associated with higher abdominal muscle density and lower abdominal muscle area (15). Such conflicting results regarding the relationship between lipid parameters and muscle area and density suggest the need for more accurate quantification of muscle quality and myosteatosis.

Although the definition of myosteatosis is not yet standardized, evaluating attenuation density and IMAT through CT is adequate for assessing myosteatosis (7, 9). NAMA has a lower level of fatty infiltration than LAMA, which has a higher quantity of adipocytes and intramyocellular fat within muscle fibers and myocytes, leading to a lower density on CT (32). Kim et al. have introduced the NAMA/TAMA index, calculated by dividing NAMA by TAMA and multiplying by 100 (23). More recent discoveries suggested the clinical value and relevance of this index; a greater NAMA/TAMA index was significantly associated with a lower prevalence of subclinical coronary artery disease and NAFLD (10, 11). The results of this study further suggest that the NAMA/TAMA index is inversely correlated with HDL-C and directly associated with LDL-C (females only) and TG.

LDL-C and sdLDL were inversely correlated with NAMA/TAMA, good quality muscle index, in women, which had been demonstrated in female patients with rheumatoid arthritis using skeletal muscle mass measured using DXA (33). Interestingly, LDL-C and sdLDL-C did not show significant associations with NAMA/TAMA in the male population. The OR of dyslipidemia according to LDL-C definition was only significant in women and did not differ according to NAMA/TAMA quartiles in men, even after adjusting for multiple variables. Hepatic lipase, a lipolytic enzyme hydrolyzing TG and phospholipid into LDL-C and HDL-C, is more active in males with higher intraabdominal fat (34). Since analysis with NAMA/TAMA index includes only inter/intramuscular fat, it might not have shown significant correlations with LDL-C and sdLDL-C in men who have more than twice the visceral fat of women (35, 36). Further studies are needed to elucidate the sex differences in the associations between myosteatosis and LDL-C.

The exact molecular mechanisms underlying the interaction between dyslipidemia and myosteatosis are not fully understood. Increased muscle fat infiltration induces lipotoxicity and chronic inflammation and results in insulin resistance in skeletal muscle (37). Insulin resistance in skeletal muscle alters the processing pattern of ingested carbohydrates by decreasing glycogen synthesis in muscle and increasing lipogenesis in the liver (38). Cytokines such as interleukin-1 and tumor necrosis factor are released, accelerating protein catabolism (39). Consequently, dyslipidemia develops with the rise in plasma TG and fall in HDL-C (38). Further research is warranted to further elucidate the underlying mechanism for the relationship between myosteatosis and dyslipidemia.

This study has several limitations. First, this is a cross-sectional study, so the causal relationship between myosteatosis and dyslipidemia could not be verified. Second, the study population was limited to Koreans, limiting its generalizability to other ethnic groups. Third, muscle quality assessed via CT scan was used to indirectly measure muscle strength without performing a grip strength test, which was not covered by routine health examinations. Lastly, the myosteatosis indices in this study were obtained solely at the L3 level of the abdomen. Although other body parts were not covered, such as the lower extremities (a common focus in previous studies), a cross-sectional area of muscle and adipose tissue from a single lumbar CT or MRI image was highly correlated with that of the whole body in several studies (40, 41). Despite such limitations, this study is the first to examine the association between myosteatosis and dyslipidemia in a large population. Previous studies measuring muscle density using CT were small population studies. Many potential confounders were considered in the statistical analysis to avoid potential bias. This research used previous abdominal CT scans taken as part of a routine health examination, so evaluation of myosteatosis in patients with a previous CT scan is clinically applicable.

Myosteatosis and dyslipidemia are closely related, evidenced by the decline in the risk of dyslipidemia as the healthy muscle component increases. Ectopic fat accumulation in muscle contributes to atherogenic dyslipidemia and ensuing cardiometabolic diseases. Future studies suggesting efficacious ways to screen and treat myosteatosis are needed.

## Data availability statement

The raw data supporting the conclusions of this article will be made available by the authors, without undue reservation.

## Ethics statement

The studies involving humans were approved by Institutional Review Board of Asan Medical Center. The studies were conducted in accordance with the local legislation and institutional requirements. The participants provided their written informed consent to participate in this study.

## Author contributions

HK: Data curation, Formal analysis, Funding acquisition, Investigation, Writing – original draft, Writing – review & editing. YC: Investigation, Methodology, Writing – review & editing. MK: Data curation, Resources, Writing – original draft. ML: Data curation, Investigation, Writing – review & editing. EK: Resources, Software, Validation, Writing – review & editing. WL: Investigation, Resources, Supervision, Validation, Writing – review & editing. HK-K: Data curation, Project administration, Resources, Supervision, Validation, Writing – review & editing. CJ: Conceptualization, Methodology, Supervision, Writing – review & editing.
