# Supplementary material for: Association between atherogenic dyslipidemia and muscle quality defined by myosteatosis
Source: Front Endocrinol (Lausanne). 2024 Aug 7;15:1327522. doi: 10.3389/fendo.2024.1327522 (PMC11335673; doi:10.3389/fendo.2024.1327522)
Supplement: Supplementary file 1 [file DataSheet_1.docx]

Supplementary Material

**Supplemental Table S1. The cut-off value for quartiles of the NAMA/TAMA index**

|  | Q1 | Q2 | Q3 | Q4 |
| --- | --- | --- | --- | --- |
| Males | ≤73.84 | 73.85–79.20 | 79.21–83.57 | ≥83.58 |
| Females | ≤67.60 | 67.61–74.42 | 74.43–79.77 | ≥79.78 |

NAMA, normal attenuation muscle area; TAMA, total abdominal muscle area; Q, quartile.

**Supplemental Table S2. Baseline characteristics of all participants**

|  | **Males** | |  | **Females** | |  |
| --- | --- | --- | --- | --- | --- | --- |
|  | **Control**  (n = 3,606) | **Dyslipidemia**  (n = 2,828) | **P-value** | **Control**  (n = 3,444) | **Dyslipidemia**  (n = 1,945) | **P-value** |
| **Anthropometric data** |  |  |  |  |  |  |
| Age, years | 53.9 ± 9.5 | 52.5 ± 8.8 | <0.001 | 51.3 ± 8.7 | 54.4 ± 8.2 | <0.001 |
| Height, cm | 170.5 ± 5.9 | 170.8 ± 5.6 | 0.053 | 159.0 ± 5.3 | 157.7 ± 5.3 | <0.001 |
| Weight, kg | 69.0 ± 9.3 | 73.5 ± 9.8 | <0.001 | 55.5 ± 7.3 | 58.8 ± 8.1 | <0.001 |
| BMI, kg/m^2^ | 23.7 ± 2.7 | 25.2 ± 2.8 | <0.001 | 22.0 ± 2.8 | 23.7 ± 3.0 | <0.001 |
| WC, cm | 84.9 ± 7.7 | 89.1 ± 7.3 | <0.001 | 76.7 ± 8.0 | 81.5 ± 7.9 | <0.001 |
| SBP, mmHg | 122.8 ± 12.9 | 125.4 ± 13.1 | <0.001 | 115.3 ± 14.2 | 120.2 ± 15.2 | <0.001 |
| DBP, mmHg | 78.2 ± 9.8 | 80.6 ± 10.4 | <0.001 | 71.8 ± 10.5 | 74.8 ± 10.7 | <0.001 |
| Skeletal muscle mass, kg | 30.6 ± 3.7 | 31.8 ± 3.8 | <0.001 | 21.5 ± 2.3 | 21.8 ± 2.5 | 0.023 |
| Body fat mass, kg | 14.4 ± 5.1 | 17.1 ± 5.4 | <0.001 | 15.8 ± 5.2 | 18.6 ± 5.5 | <0.001 |
| **Biochemical data** |  |  |  |  |  |  |
| Fasting glucose, mg/dL | 99.1 ± 16.7 | 102.8 ± 19.7 | <0.001 | 94.3 ± 13.8 | 99.5 ± 17.2 | <0.001 |
| HbA1c, % | 5.6 ± 0.6 | 5.7 ± 0.8 | <0.001 | 5.5 ± 0.5 | 5.7 ± 0.6 | <0.001 |
| Total cholesterol, mg/dL | 187.4 ± 26.4 | 205.9 ± 37.9 | <0.001 | 194.3 ± 26.8 | 212.7 ± 40.7 | 0.059 |
| Triglycerides, mg/dL | 88.0 (70.0–112.0) | 162.0 (122.0–204.0) | <0.001 | 71.0 (56.0–92.0) | 120.0 (87.0–164.0) | <0.001 |
| HDL-C, mg/dL | 55.7 ± 11.3 | 42.9 ± 9.7 | <0.001 | 68.0 ± 12.3 | 50.5 ± 11.6 | <0.001 |
| LDL-C, mg/dL | 118.5 ± 23.4 | 137.7 ± 32.4 | <0.001 | 115.8 ± 23.5 | 143.0 ± 33.8 | 0.421 |
| AST, IU/L | 25.0 (21.0–30.0) | 26.0 (22.0–32.0) | <0.001 | 22.0 (19.0–27.0) | 24.0 (20.0–29.0) | <0.001 |
| ALT, IU/L | 21.0 (16.0–29.0) | 26.0 (20.0–37.0) | <0.001 | 16.0 (13.0–21.0) | 19.0 (14.0–26.0) | <0.001 |
| hsCRP, mg/dL | 0.04 (0.02–0.10) | 0.07 (0.02–0.14) | <0.001 | 0.03 (0.02–0.06) | 0.05 (0.03–0.05) | <0.001 |
| eGFR, mL/min/1.73 cm^2^ | 93.4 ± 15.0 | 92.7 ± 15.1 | 0.083 | 102.0 ± 17.4 | 98.1 ± 17.3 | <0.001 |
| **Clinical data** |  |  |  |  |  |  |
| Current smoker, n (%) | 908 (25.2) | 1,112 (39.3) | <0.001 | 99 (2.9) | 56 (2.9) | 0.001 |
| Alcohol consumption, g/d | 0.0 (0.0–1.2) | 0.0 (0.0–1.2) | <0.001 | 0.0 (0.0–1.2) | 0.0 (0.0–1.2) | <0.001 |
| Regular exercise, n (%) | 2,312 (64.3) | 1,484 (52.5) | <0.001 | 1,949 (56.7) | 1,054 (54.4) | 0.160 |
| Menopause, n (%) | - | - | - | 1,851 (53.7) | 1,340 (68.9) | <0.001 |
| Diabetes, n (%) | 354 (9.8) | 367 (13.0) | <0.001 | 130 (3.8) | 177 (9.1) | <0.001 |
| Hypertension, n (%) | 1,113 (30.9) | 1,034 (36.6) | <0.001 | 582 (16.9) | 512 (26.3) | <0.001 |
| **CT measurement data** |  |  |  |  |  |  |
| SMA, cm^2^ | 158.8 ± 21.0 | 166.2 ± 21.7 | <0.001 | 106.2 ± 12.6 | 108.9 ± 13.5 | <0.001 |
| SMA/BMI | 6.7 ± 0.7 | 6.6 ± 0.7 | <0.001 | 4.9 ± 0.6 | 4.6 ± 0.5 | <0.001 |
| NAMA, cm^2^ | 128.6 ± 20.9 | 133.0 ± 21.6 | <0.001 | 82.2 ± 13.3 | 80.9 ± 13.7 | <0.001 |
| NAMA/BMI | 5.5 ± 0.9 | 5.3 ± 0.8 | <0.001 | 3.8 ± 0.8 | 3.5 ± 0.7 | <0.001 |
| LAMA, cm^2^ | 30.1 ± 11.1 | 33.2 ± 11.6 | <0.001 | 24.0 ± 8.7 | 28.1 ± 9.4 | <0.001 |
| LAMA/BMI | 1.3 ± 0.4 | 1.3 ± 0.4 | <0.001 | 1.1 ± 0.3 | 1.2 ± 0.3 | <0.001 |
| NAMA/TAMA index | 78.5 ± 7.9 | 77.3 ± 7.8 | <0.001 | 74.1 ± 9.3 | 70.5 ± 9.5 | <0.001 |
| VFA/SFA | 1.0 ± 0.5 | 1.2 ± 0.5 | <0.001 | 0.4 ± 0.2 | 0.6 ± 0.3 | <0.001 |

Data are presented as mean ± standard deviation or median (interquartile range, 1st–4th) unless otherwise indicated.

CT, computed tomography; BMI, body mass index; WC, waist circumference; SBP, systolic blood pressure; DBP, diastolic blood pressure; HbA1c, glycated hemoglobin; HDL-C, high-density lipoprotein cholesterol; LDL-C, low-density lipoprotein cholesterol; AST, aspartate aminotransferase; ALT, alanine aminotransferase; hsCRP, high-sensitivity C-reactive protein; eGFR, estimated glomerular filtration rate; SMA, skeletal muscle area; NAMA, normal attenuation muscle area; LAMA, low attenuation muscle area; TAMA, total abdominal muscle area; VFA, visceral fat area; SFA, subcutaneous fat area.

**Supplemental Figure S1.** **An example of body composition analysis from an axial CT slice and a schematic diagram of segmentation**


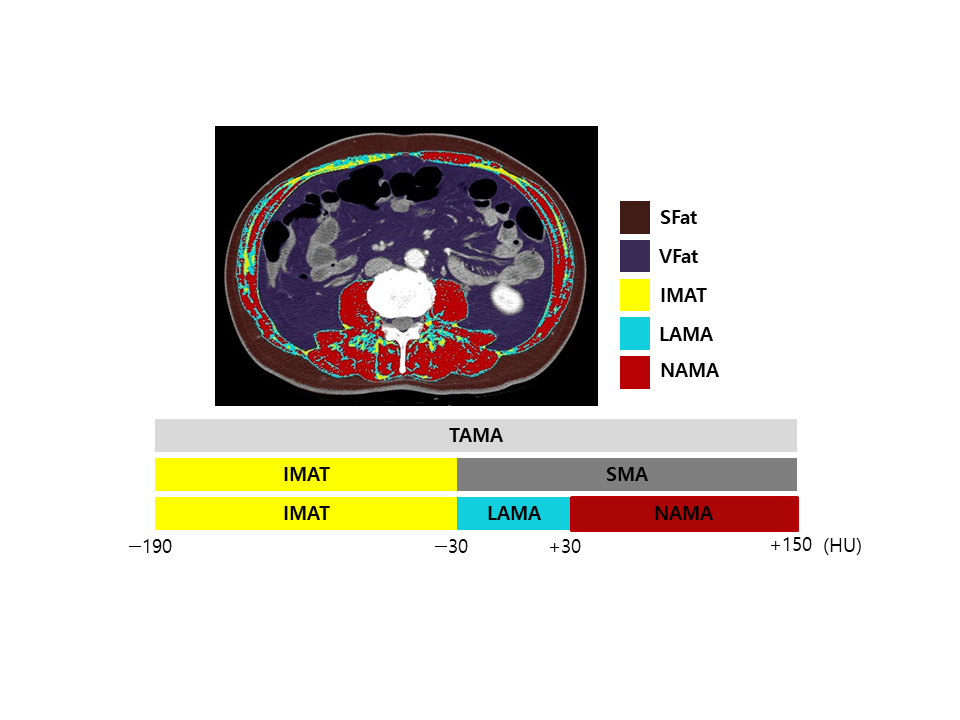


CT, computed tomography; HU, Hounsfield unit; IMAT, intramuscular adipose tissue area; LAMA, low attenuation muscle area; NAMA, normal attenuation muscle area; SFat, subcutaneous fat area; SMA, skeletal muscle area; TAMA, total abdominal muscle area; VFat, visceral fat area.
